# Dermatomyositis: Prevalence, Clinical Spectrum, Diagnostic Approach, and Management Strategies

**DOI:** 10.26502/aimr.0233

**Published:** 2026-01-28

**Authors:** Amrita Sandhu, Devendra K. Agrawal

**Affiliations:** 1 Department of Translational Research, College of Osteopathic Medicine of the Pacific, Western University of Health Sciences, Pomona, California 91766 USA

**Keywords:** Amyopathic dermatomyositis, Amyopathy, Anti-transcription intermediary factor 1-gamma, Autoantibodies, Autoimmune disorder, Dermatomyositis, Gottron’s papules, Glucocorticoids, Heliotrope rash, Immunoglobulin, Immunosuppressive therapy, Inflammation, Interstitial lung disease, Myopathy, Paraneoplastic dermatomyositis, Steroids

## Abstract

Dermatomyositis is a rare, inflammatory myopathy with signature cutaneous manifestation and variable degrees of muscular and systemic involvement. Clinical phenotypes range from muscle-predominant disease to amyopathic presentations, leading to diagnostic complexity and heterogeneity in disease trajectory. Immunologic testing reveals myositis-specific autoantibodies that associate with characteristic clinical patterns, pattern of organ involvement, and prognostic implications, including interstitial lung disease and malignancy. The absence of definitive serologic markers in all cases of dermatomyositis requires a comprehensive diagnostic approach integrating clinical features, supportive testing, and histopathologic evaluation in dermatomyositis. Current management approaches include systemic glucocorticoids, conventional and emerging immunosuppressive therapies, and intravenous immunoglobulin. Moving forward, improved understanding of disease heterogeneity and immune pathways is expected to influence personalized approaches to diagnosis and treatment in dermatomyositis. This critical review article integrates current evidence on the epidemiology, clinical presentation, diagnostic framework, systemic association, and management of dermatomyositis, highlighting ongoing challenges and future directions in the care of this intricate autoimmune disorder.

## 1. Introduction

Dermatomyositis is a complex, systemic autoimmune disorder characterized by distinctive cutaneous manifestations alongside variable degrees of inflammatory myopathy [1]. Muscular involvement typically includes symmetric, proximal muscle weakness [1,2]. Characteristic skin findings, including Gottron’s papules and heliotrope rash, serve as key diagnostic criteria for dermatomyositis and may precede or occur independently of myopathy [3,4]. Based on differences in cutaneous and muscular presentation, dermatomyositis can be categorized into four main variants: classic dermatomyositis (rash and weakness), clinically amyopathic dermatomyositis (rash without weakness), paraneoplastic dermatomyositis (associated with underlying malignancy), and juvenile dermatomyositis.

Although previously grouped with other idiopathic inflammatory myopathies, dermatomyositis is being recognized as a heterogeneous disease spectrum [6,7]. Advancements in serologic testing allow for identification of myositis-specific autoantibodies that correspond to characteristic clinical outcomes, prognosis, pattern of organ involvement, and treatment response [8]. Recognition of antibody-associated disease patterns has refined classification of dermatomyositis and provides more insight into its pathogenesis, including immune-driven inflammation and microvascular injury [1,9].

While dermatomyositis classically involves skin and muscular presentations, it is also associated with systemic complications that contribute to disease-related morbidity and mortality [10]. Based on the presence of certain autoantibodies, patients are at high risk for either interstitial lung disease or malignancy [10,11]. Understanding the relationship of dermatomyositis with systemic associations further emphasizes the importance of early diagnosis, thorough evaluation, and risk assessment [12]. This review summarizes current understanding of epidemiology, clinical presentation, serological markers, systemic associations, and treatment for dermatomyositis.

## 2. Epidemiology and Demographics of Dermatomyositis

Dermatomyositis is a rare inflammatory myopathy, with estimated reported adult incidence rates ranging from 1 to 15 cases per million population per year and a reported prevalence ranging between 2 to 20 per 100,000 individuals [1,13,14]. Dermatomyositis occurs more frequently in females, with a female-to-male ratio of approximately 2:1 [15]. Dermatomyositis demonstrates a bimodal age distribution, with incidence peaks in childhood and another in mid-to-late adulthood [1]. Since the 1940s, reported incidence rates of dermatomyositis have increased; however, true incidence trajectories remain unclear, as this trend is thought to reflect evolving study methodologies, increasingly specific diagnostic criteria, and improved disease recognition rather than significant changes in disease frequency [16]. Despite advances in disease classification and serological testing, diagnostic delay remains to be a significant challenge in clinical settings [17,18].

Dermatomyositis is considered to have a multifactorial pathogenesis, arising from the interaction of genetic susceptibility with environmental and immune triggers [6,19,20]. Infectious exposures have been proposed as potential precipitating factors, as viral pathways promote disease pathogenesis through sustained activation of innate immune pathways and type I interferon signaling, which is characteristic of dermatomyositis [21,22]. Ultraviolet radiation has also been implicated, especially in cutaneous-dominant disease presentation, where UV exposure may enhance autoantigen expression and interferon-mediated immune responses [23,24]. Drug-associated dermatomyositis has been increasingly recognized, most notably in patients treated with immune checkpoint inhibitors, which either can induce dermatomyositis or dermatomyositis-like syndromes through immune dysregulation [25].

Malignancy represents one of the strongest and most consistently reported risk factors for dermatomyositis, particularly in older adults, supporting its classification as a paraneoplastic syndrome in a subset of patients [26,27]. Dermatomyositis also coexists with other autoimmune diseases, including lupus erythematosus, autoimmune thyroid disease, and systemic sclerosis, suggesting shared immunologic susceptibility [6,28].

## 3. Clinical Presentation of Dermatomyositis

### 3a. Cutaneous Manifestations of Dermatomyositis

Dermatomyositis has a spectrum of hallmark manifestations that precede, overlap with, or occur independently of muscle involvement, and often leads to clinical evaluation [3,29,30]. The heliotrope rash, consisting of violaceous erythema of the upper eyelids with or without periorbital edema, is a recognizable dermatologic finding [15,31,32]. Gottron papules and Gottron sign, erythematous to violaceous papules or plaques over extensor surfaces of the metacarpophalangeal and interphalangeal joints, are considered pathognomonic and used to distinguish dermatomyositis from other inflammatory myopathies [32,33].

Additional cutaneous findings include photosensitive rashes involving the anterior chest (V-sign), posterior neck and shoulders (shawl sign), and lateral thighs (holster sign) are commonly observed and reflect light-induced disease activity [32,34]. Further, periungual erythema with nailfold capillary abnormalities, scalp erythema, and hyperkeratosis and fissuring of the lateral aspects of fingers and palms (mechanic’s hands) are other cutaneous features of dermatomyositis [35,36]. Calcinosis cutis, firm nodules consisting of depositions of calcium salts in the skin and subcutaneous tissues, are particularly common in juvenile dermatomyositis [37,38].

### 3b. Musculoskeletal and Systemic Findings in Dermatomyositis

Key musculoskeletal findings of dermatomyositis are symmetric, proximal muscle weakness, predominantly affecting the shoulder and pelvic girdle, leading to difficulties with activities such as climbing stairs, lifting overhead objects, and standing from a seated position [34]. Muscle weakness typically develops gradually over weeks to months, whereas muscle pain (myalgias) can occur but does not present universally [29]. Laboratory markers such as serum creatine kinase, a marker of muscle injury when elevated, demonstrate considerable variability and may be normal in select phenotypes of dermatomyositis [39,40].

Systemic findings reflect the multisystem nature of dermatomyositis and vary based on the specific disease phenotype. Dysphagia due to oropharyngeal and upper esophageal muscle involvement occurs in a significant number of patients and is associated with increased morbidity and mortality [41,42]. Cardiac involvement such as myocarditis and conduction abnormalities is detected through advanced imaging techniques rather than clinical presentation [43]. Additional systemic findings include fatigue, fever, weight loss, arthralgias, and Raynaud phenomenon [44].

### 3c. Different Phenotypes of Dermatomyositis and the Role of Autoantibodies

**Figure 1:**
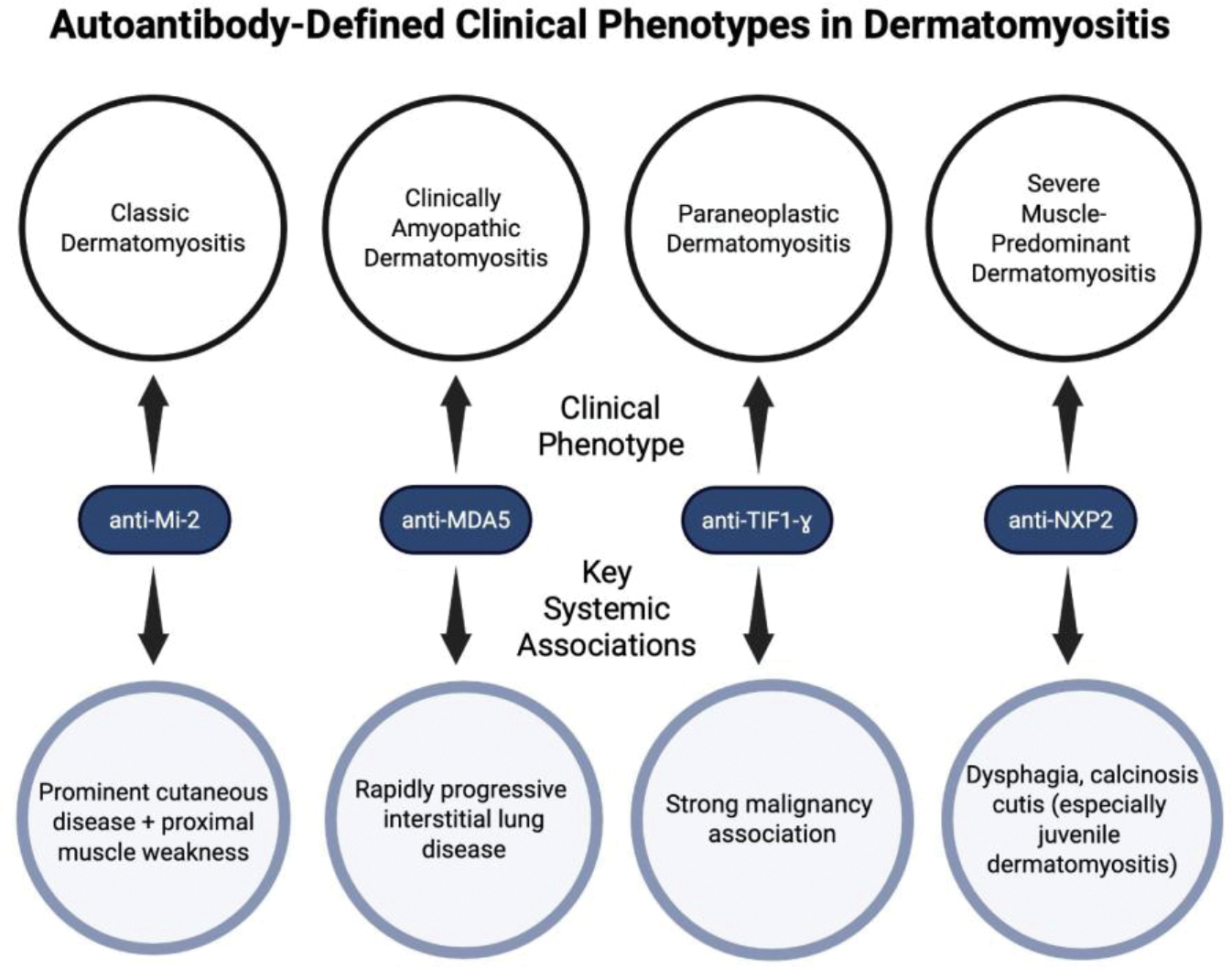
Association between myositis-specific autoantibodies and clinical phenotypes in dermatomyositis.

Dermatomyositis encompasses a heterogeneous group of clinical phenotypes that strongly correlate with characteristic myositis-specific autoantibodies, which further dictate patterns of cutaneous, muscular, and systemic involvement [45] (Figure 1).

Patients with anti-Mi-2 antibodies present with classic dermatomyositis, including both prominent cutaneous manifestations and proximal muscle weakness [46]. This phenotype of dermatomyositis has fewer high-risk complications, such as interstitial lung disease and malignancy, and shows a favorable response to therapy, resulting in improved outcomes [46].

In contrast, anti-melanoma differentiation-associated gene 5 (anti-MDA5) antibodies are commonly associated with clinically amyopathic dermatomyositis and are strongly associated with rapidly progressive interstitial lung disease (ILD) [9,47]. ILD, characterized by inflammation and fibrosis of the lung interstitium, is a leading cause of morbidity and mortality in dermatomyositis and represents one of the most severe clinical phenotypes [48].

Anti-transcription intermediary factor 1-gamma (anti-TIF1-ɣ) antibodies are present in malignancy-associated dermatomyositis in adults [49]. The strong link between the presence of TIF1-ɣ and malignancy supports a paraneoplastic phenotype of dermatomyositis, in which disease manifestations occur due to an immune-mediated or hormonal response to an underlying malignancy [50].

Further, anti-nuclear matrix protein 2 (anti-NXP2) antibodies have been linked to severe muscle involvement and dysphagia occurring across age groups, with calcinosis cutis (deposition of calcium salts in the skin and subcutaneous tissue) occurring most notably in juvenile dermatomyositis [51,52]. The presence of anti-NXP2 antibodies is associated with age-dependent differences in clinical manifestations, highlighting that the same antibody can give rise to distinct disease presentations depending on the patient’s demographics [53].

Overall, recognition of patient autoantibodies helps define the specific dermatomyositis phenotypes and is integral to characterizing clinical presentation, anticipating certain systemic findings, and guiding prognosis and risk stratification in patients with dermatomyositis (Figure 1).

## 4. Major Systemic Associations with Dermatomyositis

Dermatomyositis is a systemic autoimmune disease with extracutaneous and extramuscular presentations, most notably affecting the pulmonary system and conferring an increased risk of malignancy [54]. These systemic findings significantly contribute to disease-related morbidity and mortality, and influence both prognosis, treatment, and management strategies.

### 4a. Association of Dermatomyositis with Pulmonary Disease

Interstitial lung disease (ILD) is one of the most common and severe systemic manifestations of dermatomyositis and represents a major determinant of long-term outcomes [55]. Recent studies estimate that ILD occurs in approximately 20–45% of patients with dermatomyositis, with prevalence varying by cohort, diagnostic criteria, and autoantibody presence [56]. ILD occurs most commonly in patients with an autoantibody profile of anti-melanoma differentiation-associated gene 5 (anti-MDA5) and anti-synthetase antibodies, and can precede muscle or cutaneous symptoms or occur at any stage of dermatomyositis, and can even progress despite immunosuppressive therapy [57]. Anti-MDA5-positive dermatomyositis is frequently associated with clinically amyopathic disease and rapidly progressive ILD [58,59]. More specifically, rapidly progressive ILD represents a very severe phenotype defined by accelerated respiratory decline and high early mortality, with reported fatality rates estimating 50% within six months in certain cohorts [60]. Treatment of dermatomyositis -associated ILD requires intense immunosuppression, combining high-dose systemic glucocorticoids and immunosuppressive agents specialized to disease severity and progression [61]. Risk factors for ILD development and poorer prognosis in dermatomyositis include certain autoantibodies, elevated inflammatory markers, older age at onset, and clinical symptoms such as fever [59].

### 4b. Association of Dermatomyositis with Malignancy

Dermatomyositis is strongly associated with malignancy, particularly in the general adult populations, and is categorized as a paraneoplastic syndrome in certain subsets of dermatomyositis [62]. Large epidemiological studies and meta-analyses demonstrate that patients with dermatomyositis have a significantly higher risk of malignancy, with the highest risk occurring within three years before or after disease diagnosis [61]. A wide spectrum of malignancies are associated with dermatomyositis, including ovarian, breast, colorectal, pancreatic, lung, and hematologic cancers, and specific myositis-associated autoantibodies such as anti-TIF1ɣ increases malignancy risk [61]. In paraneoplastic dermatomyositis, treatment of the underlying cancer can lead to improvement or resolution of myositis symptoms, typically in conjunction with immunosuppressive therapy [63]. Utilizing screening strategies specialized for high-risk dermatomyositis patients, particularly for patients of older age at disease onset, male sex, dysphagia and systemic inflammation, can facilitate earlier detection of malignancy [64].

## 5. Diagnosis of Dermatomyositis

**Figure 2:**
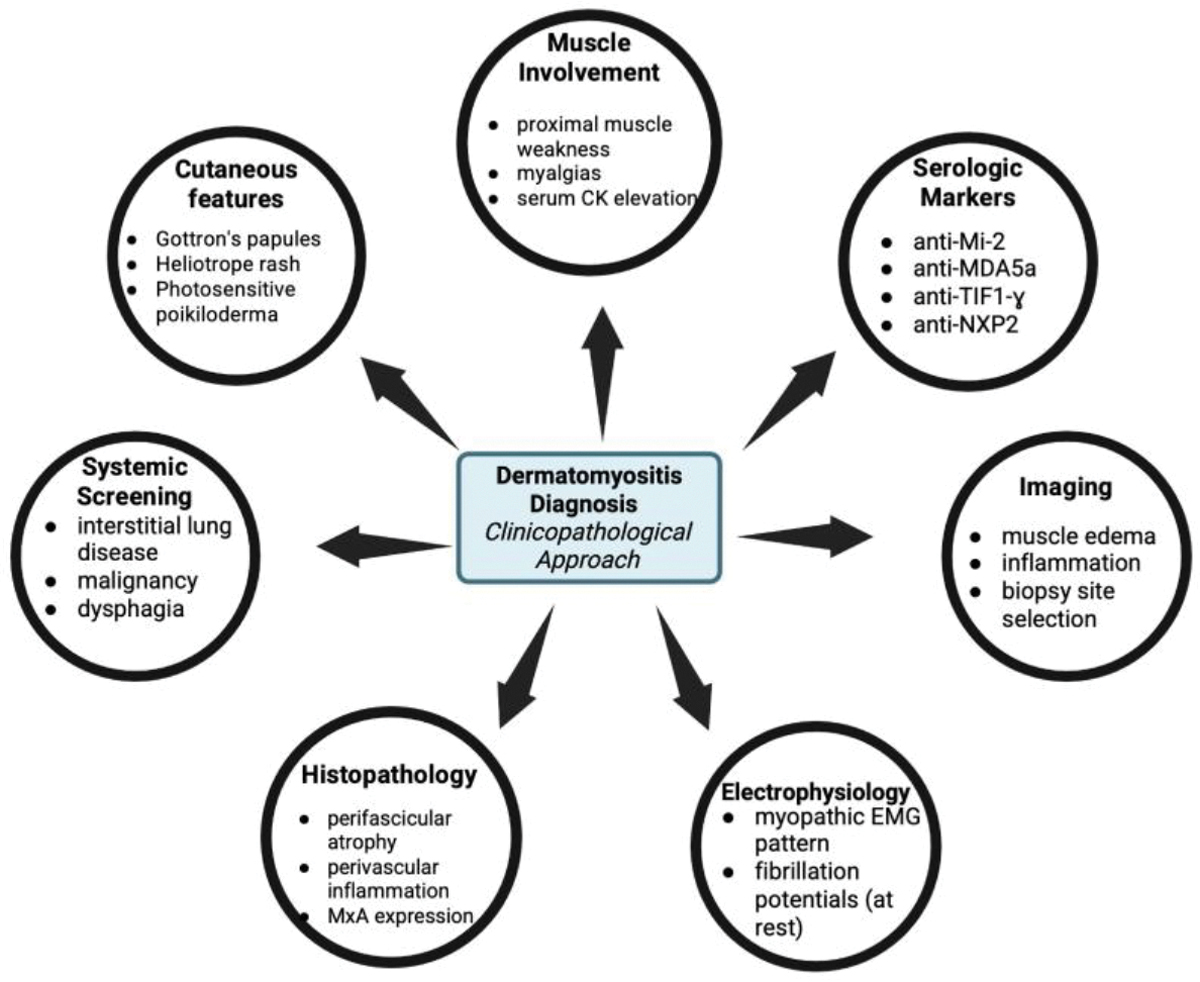
Integrated diagnostic framework for dermatomyositis. An integrated clinicopathological approach supports accurate diagnosis, phenotype classification, and prognostic evaluation.

Diagnosis of dermatomyositis utilizes a clinicopathological approach, integrating clinical manifestations, laboratory testing, serologic markers, imaging, electromyographic and histopathologic data (Figure 2) [65]. The 2017 EULAR/ACR classification criteria use weighted clinical, laboratory, and histopathological features to classify dermatomyositis within the spectrum of idiopathic inflammatory myopathies, with increasing sensitivity when a muscle biopsy is performed [66].

Since dermatomyositis presentation varies from clinically amyopathic phenotypes to muscle-predominant disease, diagnostic evaluation should be tailored according to the patient’s symptoms and systemic risk profile. Clinically, hallmark cutaneous findings such as Gottron’s papules over extensor joints and periorbital heliotrope rash are highly indicative of dermatomyositis and support diagnosis in the absence of muscle symptoms [67]. Additional characteristic cutaneous findings include photosensitive poikiloderma including the anterior chest (V-sign), posterior neck and shoulders (shawl sign), and lateral thighs (holster sign), as well as periungual erythema with nailfold capillary abnormalities affecting the fingers and, less commonly, the toes [68]. Proximal muscle weakness can also be suggestive of dermatomyositis [69]. Upon initial evaluation, screening for systemic manifestations such as interstitial lung disease, malignancy, and dysphagia should occur simultaneously, as that can influence urgency and prognosis [70]. Laboratory tests assessing elevated serum muscle enzymes such as creatine kinase, aldolase, and lactate dehydrogenase are frequently observed [34]. However, such elevations do not solely indicate dermatomyositis since similar abnormalities can occur in other myopathies [34]. Conversely, normal enzyme levels do not exclude dermatomyositis as a differential diagnosis, especially in phenotypes such as clinically amyopathic dermatomyositis [34]. Since dermatomyositis can present without clinically apparent weakness, objective and repeated assessments (muscle strength and laboratory testing) should be performed to distinguish between clinically amyopathic dermatomyositis from early myositis [71].

Serologic testing for myositis-specific antibodies (MSA), such as anti-Mi-2, anti-MDA5, anti-TIFɣ, anti-NXP2, and others can aid in diagnosis and helps define specific subtypes of dermatomyositis. However, the presence of autoantibodies alone is not enough to confidently diagnose dermatomyositis, and must be interpreted in a clinical context [72,73]. Seronegative dermatomyositis is very common, especially in cutaneous dominant phenotypes where only characteristic rashes are present [74]. There is not one specific serological marker that is pathognomonic for dermatomyositis, and even highly associated autoantibodies (anti-Mi-2, anti-MDA5, etc.) are utilized for diagnostic support rather than sole diagnostic proof [7].

In regards to imaging, MRI is commonly used as a non-invasive tool to detect muscle edema and inflammation, and can pinpoint muscle biopsy sites [75,76]. Electromyography (EMG), a diagnostic test that evaluates muscle function by measuring muscular electrical activity, can demonstrate a myopathic pattern with fibrillation potentials at rest and short-duration, low-amplitude motor unit potentials with voluntary contraction [77]. EMG can be beneficial for selecting an appropriate muscle for biopsy, but it is limited by its lack of specificity and invasive nature [77]. Muscle biopsy can provide histopathologic support of dermatomyositis, particularly revealing perifascicular atrophy, perivascular inflammation, perifascicular myxovirus resistance protein A (MxA) expression, and capillary abnormalities [74,78]. There is increasing support for MxA overexpression being pathologically characteristic of dermatomyositis, rather than other inflammatory and noninflammatory myopathies [74,78]. Skin biopsies can also be utilized to support dermatomyositis diagnosis when cutaneous symptoms are present with minimal muscular findings, but results should be interpreted in the context of clinical features and serology [34]. To obtain the most accurate diagnosis of dermatomyositis, hallmark cutaneous findings, evidence of muscle involvement, and supportive data from clinical examination, laboratory testing, imaging (MRI/EMG), histopathology, and myositis-specific autoantibodies should be integrated to define the specific phenotype and adjust prognostic assessment [79].

## 6. Management and Treatment of Dermatomyositis

Management of dermatomyositis requires a multidisciplinary approach, influenced by the dominant disease manifestation (skin, muscle, systemic symptoms), disease severity, and autoantibody-associated risk features [80–82]. Current guidelines emphasize suppressing inflammation with both nonpharmacologic measures and systemic therapy, thereby improving cutaneous disease, muscle strength, and preventing irreversible organ damage [80–82]. For phenotypes with predominantly cutaneous symptoms, treatment involves photoprotection, topical corticosteroids/topical calcineurin inhibitors, and systemic modulators when topical therapy is insufficient [24,80].

### 6a. Current Treatment of Dermatomyositis (General Approach)

Systemic glucocorticoids remain the first-line treatment for dermatomyositis due to its fast-acting anti-inflammatory effects and efficacy in improving muscle strength [83]. However, due to detrimental effects of long-term steroid use, it is recommended to utilize steroids in limited durations and incorporate steroid-sparing immunosuppressive therapies [80,81]. Common non-corticosteroid immunosuppressive agents include methotrexate, azathioprine, and mycophenolate mofetil, with selection influenced by organ involvement, comorbidities, and tolerability [80,81]. With increasing evidence from randomized controlled trials, intravenous immunoglobulin (IVIG), a treatment involving administration of antibodies (IgG) to neutralize pathogenic autoantibodies and modulate inflammatory pathways, has emerged as a considerable option for moderate-to-severe dermatomyositis [84–86].

### 6b. Specific Treatments with Corresponding Adverse Effects

Initially, high-dose systemic glucocorticoids are often used to treat dermatomyositis, but clinicians must be aware of predictable adverse effects including metabolic and endocrine consequences, osteoporosis, hypertension, increased infection risk, and steroid-induced myopathy [80,81]. Alternatively, steroid-sparing agents are also administered including methotrexate and azathioprine, which control muscle and skin symptom progression, but have secondary effects of cytopenias and increased infection risk [80,87]. Mycophenolate mofetil is more frequently administered when interstitial lung disease is suspected or present, however notable adverse effects include gastrointestinal intolerance, infection risk, and leukopenia [81]. Most importantly, methotrexate and mycophenate mofetil are effective steroid-sparing medications, but are contraindicated in pregnancy because of teratogenic risk, with mycophenolate mofetil requiring strict contraception counseling [88].

For severe or rapidly progressive interstitial lung disease presentations, ambitious strategies include utilizing calcineurin inhibitors, including tacrolimus and cyclosporine, and/or cyclophosphamide as part of multi-agent immunosuppressive therapy [80]. Drawbacks of calcineurin inhibitors include development of hypertension, nephrotoxicity, neurotoxicity, while cyclophosphamide has adverse outcomes involving infection susceptibility, infertility, cytopenias, and even malignancy requiring careful monitoring [6].

IVIG is gaining increased support through a phase 3 randomized controlled trial in adult dermatomyositis, and is utilized when managing skin and muscle symptoms with first-line agents are ineffective or when steroid-sparing immunosuppressive therapy is needed [84–86]. While IVIG is well tolerated, there are important secondary consequences including thromboembolic events and renal complications in vulnerable patients [85,89]. Octagam 10%, the only IVIG product with FDA approval for adult dermatomyositis, has gained increased clinical recognition and insurance coverage [90].

Rituximab has an off-label use to treat dermatomyositis, especially in cases where primary therapy measures are insufficient or poorly tolerated and autoantibodies are present [91,92]. Due to Rituximab’s predominant mechanism of depleting B cells, serious implications include hypogammaglobulinemia, infection susceptibility (notably, hepatitis B reactivation), and diminished vaccine response [91].

Another treatment strategy incorporated for refractory phenotypes are Janus Kinase (JAK) inhibitors [93,94]. Prospective clinical trial data supports the clinical benefit of tofacitinib, particularly for cutaneous phenotypes; however, its use is associated with risks including thrombosis, cardiovascular events, and malignancy warnings for susceptible populations, necessitating appropriate therapeutic trade-off considerations [93,94].

### 6c. Challenges to Treatment of Dermatomyositis

Clinical challenges in the management of dermatomyositis are largely due to its disease heterogeneity, organ-dominant phenotypes, and the existence of refractory muscle or cutaneous disease despite severe immunosuppression [95]. A significant challenge is balancing the aggressive immunosuppression required for systemic presentations such as interstitial lung disease against infection susceptibility and cumulative toxicity, especially with multi-agent therapies. Another challenge presents with cutaneous disease activity following an independent journey of muscle involvement and may persist despite treatment and control of myositis, requiring specific skin-directed regimens beyond muscle-directed therapies [24].

**Figure 3:**
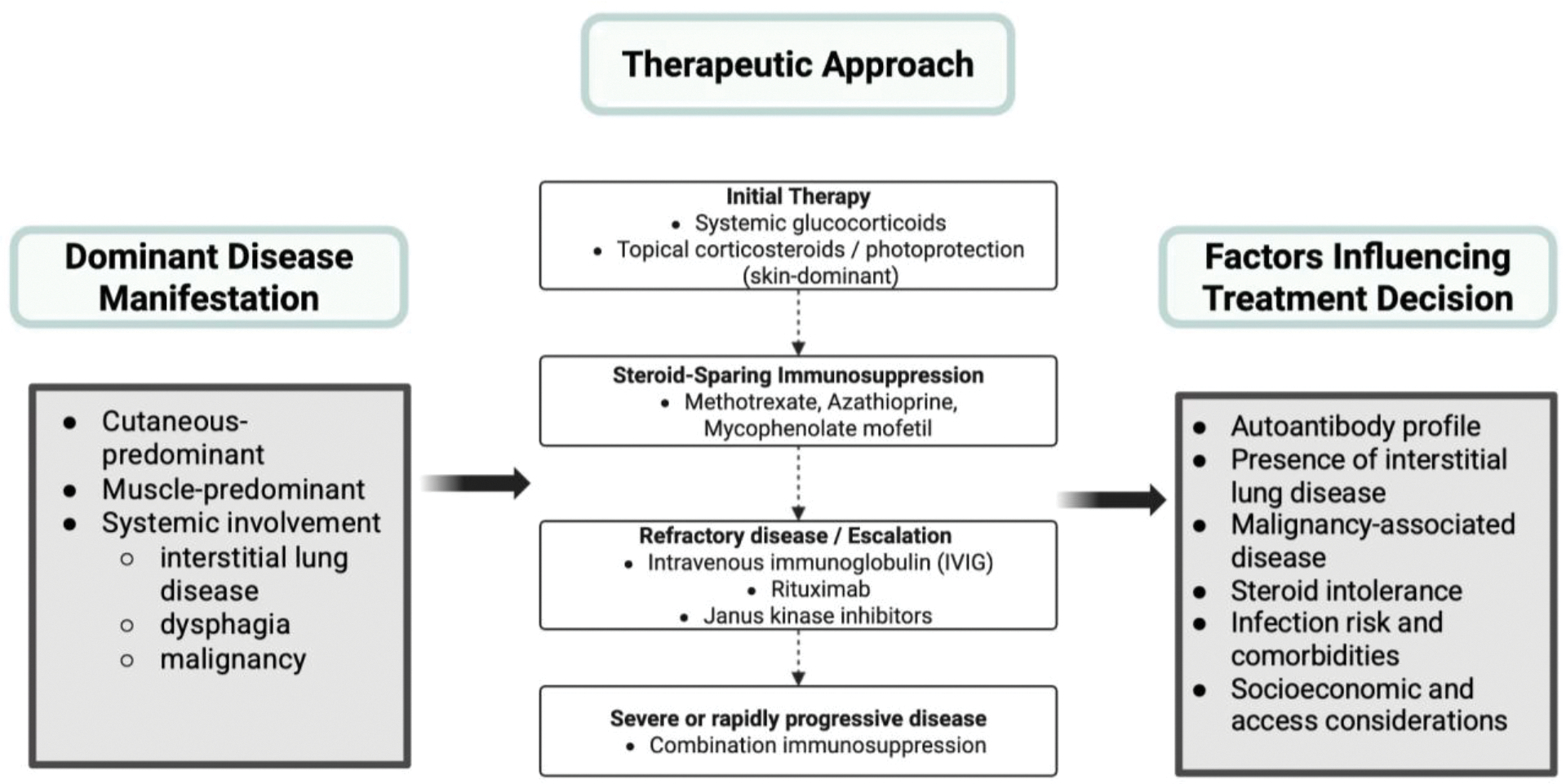
Therapeutic decision-making methodological model in dermatomyositis. While systemic glucocorticoids remain first-line therapy, steroid-sparing immunosuppressive agents, intravenous immunoglobulin, and targeted immunomodulatory therapies are utilized based on disease phenotype, treatment response, and patient-specific factors.

Socioeconomic barriers also dictate proper management of dermatomyositis, particularly limited access to specialty care, insurance coverage variation, and high medication costs (IVIG and biologics) can limit swift access and continuity of treatment [96,97]. Recent studies emphasize insurance-related differences in healthcare utilization and medication management in dermatomyositis, further supporting that access and cost factors influence access to treatment [97]. Accordingly, therapeutic decision-making necessitates intentional integration of efficacy, safety, patient comorbidities, and socioeconomic factors to optimize long-term outcomes in the treatment of dermatomyositis. Overall, management of dermatomyositis requires a personalized, phenotype-driven therapeutic approach that integrates disease severity, systemic involvement, and patient-specific risk factors (Figure 3).

## 7. Outstanding Questions and Further Research

While there has been immense progress in diagnostic methods and understanding of dermatomyositis, significant gaps persist in the identification of robust biomarkers, prognostication, and treatment optimization leading to continuous research into novel biomarkers and targeted immunotherapies.

### Discovery of New Biomarkers (Antibodies/Cytokines)

Despite identification of myositis-specific autoantibodies and its correlation to specific phenotypes of dermatomyositis, a significant number of patients remain seronegative, requiring the need for the additional biomarkers to assist in diagnosis and prognostic stratification. Recent studies have focused on expanding the range of detectable autoantibodies and optimizing diagnostic test accuracy and reliability to improve accurate disease identification across diverse patient demographics [98,99].

Beyond autoantibodies, detecting cytokine and interferon-related biomarkers is gaining increased recognition, emphasizing the pivotal role of type I interferon signaling in dermatomyositis pathogens [100,101]. Recent proteomic and immunologic studies have identified candidate biomarkers such as chemokines CXCL10 and CXCL11 correlating to immune activation and dermatomyositis progression, and can help identify a subgroup of patients who are more likely to develop interstitial lung disease [102]. Additional biomarkers include plasma proteins such as KRT19 that are significantly elevated in anti-MDA5-positive dermatomyositis patient subgroups, and may have diagnostic weight [103]. Levels of type I and type III interferons (IFN-β, IFN-γ3) are elevated in anti-MDA5-positive dermatomyositis and are associated with aggressive phenotypes such as rapidly progressive interstitial lung disease, supporting the use of interferon markers to inform risk stratification [104]. Clinical translation of these molecular insights will require validation in larger, diverse patient cohorts through randomized controlled trials, as well as the development of standardized assays suitable for routine clinical use [105].

Alongside advances in biomarker research, immunotherapy-focused investigations are expanding therapeutic options in managing dermatomyositis. Janus Kinase inhibitors and biologic medications have shown clinical improvement in dermatomyositis in small cohorts and case reports, particularly in refractory amyopathic dermatomyositis-associated ILD; however, large cohort studies are needed to generate substantial evidence [106]. In a small observational study, JAK inhibitor agents such as baricitinib have shown improvement in lung opacities and clinical outcomes [107]. The development of targeted immunomodulatory strategies, such as agents affecting interferon pathways or other critical immune pathways, is in early clinical evaluation, with Phase 2 and Phase 3 trials evaluating therapies including IFN-β neutralizing antibodies [108].

Together, these research efforts underscore the potential for integrating molecular biomarkers with immunotherapeutic approaches to improve diagnosis, prognostication, and personalized treatment in dermatomyositis. However, continued validation and robust clinical trials are required to translate these findings into practice.

## 8. Conclusion

Dermatomyositis is a multidimensional, heterogeneous autoimmune disease characterized by diverse cutaneous, muscular, and systemic presentations, with long-term outcomes strongly influenced by autoantibody profiles. Identification of myositis-specific autoantibodies has defined distinct dermatomyositis phenotypes, enabling improved prognostic evaluation for malignancy and interstitial lung disease. While significant progress has been made in diagnostic criteria and serologic testing, timely diagnosis of dermatomyositis remains challenging due to its clinical heterogeneity. Accurate diagnosis, particularly in seronegative or clinically amyopathic cases, requires a clinicopathological framework, with synthesis of clinical findings alongside laboratory, imaging, electrophysiologic and histopathologic data. High-dose glucocorticoids remain the first-line treatment; however, there is increasing support for steroid-sparing immunosuppressive and targeted therapies to balance long-term steroid-related toxicity with disease management. Therapeutic innovation in dermatomyositis, including intravenous immunoglobulin, Janus kinase inhibitors, and interferon-targeted immunomodulators, has expanded treatment options for refractory disease but requires stronger evidence from randomized controlled trials. Ongoing research aims to identify reliable biomarkers, refine antibody-based phenotypes, and advance immunomodulatory mechanisms to enhance personalized treatment and improve long-term outcomes for patients with dermatomyositis.

## Key Points

1. Dermatomyositis is a rare, systemic autoimmune disease characterized by signature cutaneous features and variable degrees of muscular symptoms.
2. Dermatomyositis represents a heterogeneous disease spectrum, including classic dermatomyositis, clinically amyopathic dermatomyositis, paraneoplastic dermatomyositis, and juvenile dermatomyositis, contributing to its diagnostic complexity.
3. Clinically amyopathic and seronegative phenotypes are gaining increased recognition and continue to be a substantial source of diagnostic delay.
4. Gottron’s papules and heliotrope rash are pathognomonic cutaneous findings and may support diagnosis in the absence of muscular manifestations.
5. Myositis-specific autoantibodies correlate with distinctive dermatomyositis phenotypes and provide clinically significant information regarding prognosis and systemic vulnerability.
6. Certain autoantibodies strongly associate with distinctive systemic manifestations, including interstitial lung disease and malignancy, which are major contributors to morbidity and mortality in dermatomyositis.
7. No single clinical presentation, biomarker, or diagnostic test is adequate to definitively diagnose dermatomyositis in isolation, highlighting an integrated clinicopathologic approach.
8. Systemic glucocorticoids remain the initial management strategy but are limited by their long-term toxicity.
9. With moderate-to-severe cases of dermatomyositis or refractory disease, steroid-sparing immunosuppressive agents and intravenous immunoglobulin are key components to management.
10. Active research efforts aim to identify robust biomarkers and develop immune-targeted therapies to enhance prognostic assessment and support individualized treatment.
